# Enteropathogens and Rotavirus Vaccine Immunogenicity in a Cluster Randomized Trial of Improved Water, Sanitation and Hygiene in Rural Zimbabwe

**DOI:** 10.1097/INF.0000000000002485

**Published:** 2019-11-15

**Authors:** James A. Church, Elizabeth T. Rogawski McQuade, Kuda Mutasa, Mami Taniuchi, Sandra Rukobo, Margaret Govha, Benjamin Lee, Marya P. Carmolli, Bernard Chasekwa, Robert Ntozini, Monica M. McNeal, Lawrence H. Moulton, Beth D. Kirkpatrick, Jie Liu, Eric R. Houpt, Jean H. Humphrey, James A. Platts-Mills, Andrew J. Prendergast

**Affiliations:** From the * Zvitambo Institute for Maternal and Child Health Research, Harare, Zimbabwe; † Centre for Genomics & Child Health, Blizard Institute, Queen Mary University of London, London, United Kingdom; ‡ Division of Infectious Diseases and International Health, University of Virginia, Charlottesville, Virginia; § Vaccine Testing Center, College of Medicine, University of Vermont, Burlington, Vermont; ¶ Department of Pediatrics, University of Cincinnati College of Medicine, Cincinnati, Ohio; ‖ Division of Infectious Diseases, Cincinnati Children’s Hospital Medical Center, Ohio; ** Department of International Health, Johns Hopkins Bloomberg School of Public Health, Baltimore, Maryland.

**Keywords:** infants, oral vaccine, Rotarix, water, sanitation and hygiene, enteropathogen

## Abstract

Oral rotavirus vaccines (RVVs) are less efficacious in low-income versus high-income settings, plausibly due to more enteropathogen exposure through poor water, sanitation and hygiene (WASH). We explored associations between enteropathogens and RVV immunogenicity and evaluated the effect of improved WASH on enteropathogen carriage.

**Methods:**

We detected stool enteropathogens using quantitative molecular methods and measured anti–rotavirus immunoglobulin A by enzyme-linked immunosorbent assay in infants enrolled to a cluster randomized 2 × 2 factorial trial of improved WASH and improved infant feeding in Zimbabwe (NCT01824940). We used multivariable regression to explore associations between enteropathogens and RVV seroconversion, seropositivity and geometric mean titer. We evaluated effects of improved WASH on enteropathogen prevalence using linear and binomial regression models with generalized estimating equations.

**Results:**

Among 224 infants with enteropathogen and immunogenicity data, 107 (47.8%) had ≥1 pathogen and 39 (17.4%) had ≥2 pathogens detected at median age 41 days (interquartile range: 35–54). RVV seroconversion was low (23.7%). After adjusting for Sabin-poliovirus quantity, pan-enterovirus quantity was positively associated with RVV seroconversion (relative risk 1.61 per 10-fold increase in pan-enterovirus; 95% confidence interval: 1.35–1.91); in the same model, Sabin quantity was negatively associated with RVV seroconversion (relative risk: 0.76; 95% confidence interval: 0.60–0.96). There were otherwise no meaningful associations between individual or total pathogens (bacteria, viruses, parasites or all pathogens) and any measure of RVV immunogenicity. Enteropathogen detection did not differ between randomized WASH and non-WASH groups.

**Conclusions:**

Enteropathogen infections were common around the time of rotavirus vaccination in rural Zimbabwean infants but did not explain poor RVV immunogenicity and were not reduced by a package of household-level WASH interventions.

The introduction of oral rotavirus vaccination has been a key factor in reducing the global burden of childhood diarrhea. However, rotavirus remains the leading cause of diarrheal mortality among children under 5 years of age.^1^ Moreover, studies consistently show that oral rotavirus vaccines (RVVs) are less efficacious in low-income compared with high-income settings.^2^ In sub-Saharan Africa, RVV efficacy against severe rotavirus gastroenteritis was only 39%^3^ and in South Asia 48%,^4^ compared with 85%–98% in Europe and United States.^5,6^ Although our understanding of oral vaccine failure remains incomplete, intestinal factors are believed to be important,^7^ including a high burden of enteropathogens in early infancy in low-income settings.^8^

Enteric infections around the time of vaccination might impede oral vaccines directly, through competition for receptor binding and cell entry, or indirectly through induction of innate immunity, thereby hampering vaccine replication.^9^ One of the clearest examples of biologic interference is the association between non-polio enteroviruses (NPEV) and reduced oral polio vaccine (OPV) seroconversion.^10^ Similarly, coadministration of 2 live vaccines (OPV and rotavirus) reduced RVV immunogenicity in trials from multiple countries.^11^ Two previous studies from Asia have explored associations between enteropathogen carriage and immune responses to RVV^12,13^; however, to our knowledge, there have been no studies from sub-Saharan Africa.

We recently reported findings from a cluster randomized trial of improved water, sanitation and hygiene (WASH) in rural Zimbabwe. Infants in the WASH arms of the trial, compared with the non-WASH arms, had a 50% increase in RVV seroconversion (from approximately 20%–30%).^14^ We hypothesized that the WASH intervention reduced enteropathogen carriage, thereby increasing RVV immunogenicity. To explore this, we used quantitative molecular methods to identify enteropathogens in stool specimens to determine (1) the association between enteropathogens and RVV immunogenicity and (2) the effect of improved WASH on enteropathogen carriage around the time of rotavirus vaccination.

## MATERIALS AND METHODS

### Study Participants

The Sanitation Hygiene Infant Nutrition Efficacy (SHINE) trial was a 2 × 2 factorial cluster randomized trial assessing the independent and combined effects of improved WASH and improved infant and young child feeding (IYCF) on stunting and anemia (NCT01824940). The study design has been described elsewhere.^15^ Briefly, women becoming pregnant between November 2012 and March 2015 were eligible if they lived in clusters randomized to standard-of-care (SOC), IYCF, WASH or combined IYCF plus WASH. In the SOC arm, village health workers promoted early, exclusive and prolonged breast-feeding together with family planning, prevention of mother-to-child HIV transmission and infant immunization. WASH households, in addition to SOC interventions, had a pit latrine^16^ and 2 hand-washing stations^17^ installed at approximately 20 weeks’ gestation, and received monthly liquid soap, water chlorination solution and an infant play-space to separate children from animals. In the IYCF arm, women received all SOC interventions plus nutrition education and small-quantity lipid-based nutrient supplement for infants between 6 and 18 months of age. Women in the IYCF plus WASH arm received all interventions. The trial found a modest effect of the IYCF intervention on linear growth and hemoglobin at 18 months of age but no effect of WASH on either primary outcome.^18^ Neither intervention reduced diarrhea.

### Rotavirus Vaccination

In May 2014, oral monovalent RVV Rotarix^TM^ (GSK Biologicals, Rixensart, Belgium) was introduced in Zimbabwe and given with OPV at 6 and 10 weeks of age. OPV (Sabin strain) is not routinely given at birth in Zimbabwe. Vaccination was undertaken at local clinics and not overseen by the trial; however, national rotavirus vaccination coverage in 2015–2016 was 87%–91%.^19^ Trial staff recorded vaccination dates by reviewing child health cards. Each child’s final Rotarix vaccination status was categorized as complete (2 doses), incomplete (1 dose) or no vaccine (zero doses).

### Substudy Population

From June 2014, infants were enrolled in a substudy with longitudinal specimen collection (including plasma and stool) at 1, 3, 6, 12 and 18 months of age.^20^ For the current analysis, infants from the substudy were eligible if they were HIV-unexposed, had at least 1 plasma sample collected post-rotavirus vaccination and an available stool sample collected around the time of the first Rotarix dose. We permitted a 30-day window of stool collection pre- or postvaccination, hypothesizing that enteric infections before, during or soon after vaccination may contribute to vaccine interference. Infants were excluded if they had missing rotavirus vaccination data, had not received at least 1 dose of RVV or had invalid stool polymerase chain reaction (PCR) results. Where >1 stool sample was available for a given child, the sample closest to the date of the first RVV dose was selected. Diarrheal stool samples were not included.

### Anti–rotavirus Immunoglobulin A Assay

Plasma anti–rotavirus immunoglobulin A (IgA) was measured by enzyme-linked immunosorbent assay in the Zvitambo laboratory, using methods previously described.^21^ Anti–rotavirus IgA is the most widely used marker of oral rotavirus vaccination or natural infection.^22^ Our primary outcome was seroconversion, defined as a postvaccine plasma concentration of anti–rotavirus IgA ≥20 U/mL in infants who were seronegative (<20 U/mL) prevaccination.^23^ Secondary outcomes included anti–rotavirus IgA titer and seropositivity, defined as a postvaccine titer ≥20 U/mL, regardless of prevaccine titer. We refer to these 3 outcomes collectively as RVV immunogenicity. The assay lower limit of detection was 7.5 U/mL as determined by the assay developers at Cincinnati Children’s Hospital Medical Center.

### Molecular Enteropathogen Detection

Stool specimens from the 1- and 3-month visits were tested at the University of Virginia using custom-developed TaqMan Array Cards (ThermoFisher, Carlsbad, CA), which compartmentalize probe-based quantitative PCR (qPCR) assays for 29 enteropathogens.^24^ Assay validation, nucleic acid extraction, qPCR conditions and quality control have been previously described.^25,26^ Pathogen quantities were defined by log_10_-copy numbers per gram of stool based on the qPCR cycle threshold. In addition, stools were tested using a cognate pan-enterovirus (EV) real-time qPCR; EV-positive stools were further tested by multiplex real-time qPCR to identify Sabin polioviruses. EV-positive but Sabin-negative stools were inferred to be NPEV, following previous approaches.^27^

### Statistical Analysis

First, we investigated whether individual enteropathogens were associated with RVV immunogenicity. All enteropathogens with prevalence ≥1% were included and detection was categorized as yes or no (binary variable), based on a qPCR cycle threshold <35 (the analytic limit of detection).^25^ Stool detection of rotavirus was excluded from these analyses because we could not distinguish wild-type and vaccine strains. Second, for EVs alone, we also estimated associations between Pan-EV quantity (continuous variable) and RVV immunogenicity adjusting for Sabin quantity to further explore the impact of NPEV on vaccine responses. We used multivariable generalized estimating equations to account for within-cluster correlation with a log-binomial link to estimate relative risks (RRs) of rotavirus seroconversion and seropositivity and an identity link to estimate mean difference in anti–rotavirus IgA geometric mean titers (GMTs). To handle zero-inflated semicontinuous GMT data, we employed a log-normal censored regression model (Tobit), with left censoring at 7.5 U/mL. Models were adjusted for birth weight, infant age at vaccination, exclusive breast-feeding status at 3 months, maternal mid-upper arm circumference, detection of other enteropathogens, birth season, concurrent OPV receipt and randomization group, which were selected a priori based on biologic plausibility.

Next, using similar methods, we assessed the relationship between overall pathogen burden and RVV immunogenicity. We expressed pathogen burden as the total number of pathogens (excluding rotavirus and poliovirus) and the total number of bacteria, viruses or parasites. We also explored mixed infection, which we defined as detection of ≥2 different pathogen classes.

We conducted sensitivity analyses to account for variation in timing of stool collection, restricting analyses to stools collected 14 days before the first RVV dose.

Finally, we evaluated the effects of improved WASH on enteropathogen prevalence around the time of RVV receipt. Analyses were intention-to-treat with exposure defined by the randomized interventions. Because the IYCF intervention started at 6 months of age (beyond the window for rotavirus vaccination and stool collection), we compared the WASH group (WASH and IYCF + WASH arms) and non-WASH group (SOC and IYCF arms). We used log-binomial regression for individual pathogens and Poisson regression for pathogen burden with generalized estimating equations and robust variance to account for within-cluster correlation. These models were adjusted for intervention arm and age at vaccine receipt.^18^

We accounted for multiple comparisons using the Benjamini–Hochberg procedure to determine false discovery rate–adjusted *P* values.^28^ Statistical analyses used STATA v14 (StataCorp LP, College Station, TX) and Prism v7 (GraphPad Software Inc., San Diego, CA).

### Ethics

Both the SHINE trial and this laboratory substudy were approved by the Medical Research Council of Zimbabwe and the Johns Hopkins Bloomberg School of Public Health Committee on Human Research. Written informed consent was obtained from all caregivers before enrolment in the trial.

## RESULTS

Among 5280 pregnant women, there were 3989 live-born HIV-unexposed infants (see Fig., Supplemental Digital Content 1, http://links.lww.com/INF/D653). Eight hundred eighty-two infants had rotavirus immunogenicity measured and 224 (25.4%) of these had stool collected within 30 days of Rotarix receipt with valid stool qPCR results. Table 1 outlines the characteristics of the 224 infants, together with maternal and household variables. Characteristics were broadly similar to live-born HIV-unexposed infants not included in this analysis (see Table, Supplemental Digital Content 2, http://links.lww.com/INF/D654). Two hundred eighteen of 224 (97.3%) infants had documented receipt of 2 RVV doses. The median age at vaccination was 6.3 weeks [interquartile range (IQR): 6.0–6.7] for dose 1 and 10.9 weeks (IQR: 10.1–11.7) for dose 2. The median timing of pre-RVV titer measurement was 9 days (IQR: 6–14) before the first dose and for post-RVV titer measurement was 27 days (IQR: 19–41) after the last dose. The median infant age at stool collection was 41 days of age (IQR: 35–54), corresponding to a median of 4 days (IQR: 10 to −7) before the first dose of RVV (see Fig., Supplemental Digital Content 3, http://links.lww.com/INF/D655). There were no significant differences in infant characteristics between seropositive and seronegative children (see Tables, Supplemental Digital Contents 4 and 5, http://links.lww.com/INF/D656 and http://links.lww.com/INF/D657).

**TABLE 1.**
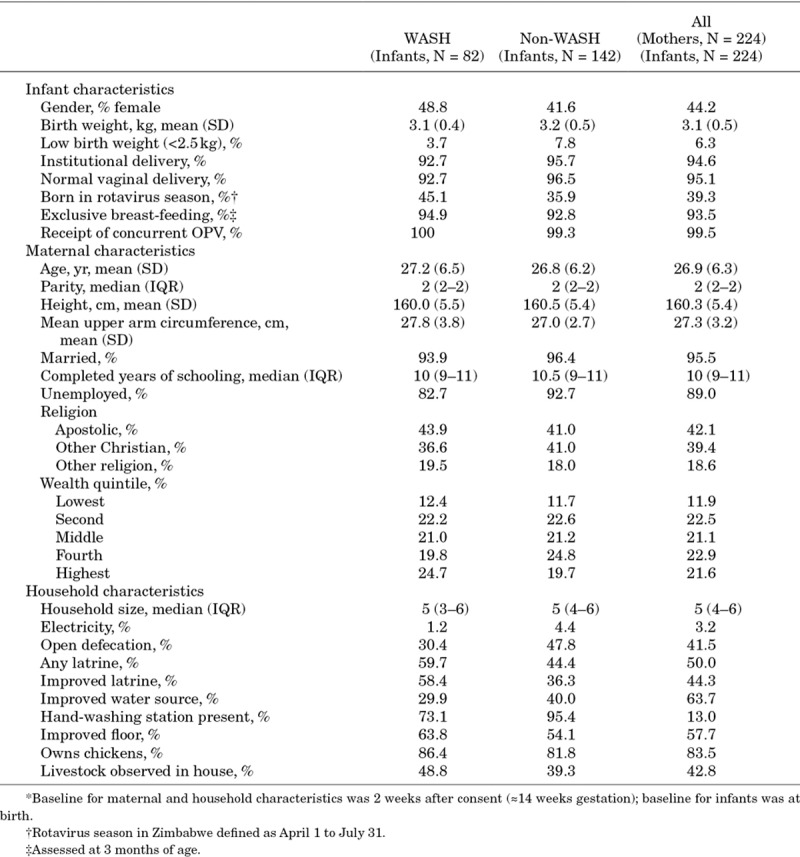
Characteristics of Infants, Mothers and Their Households*

### Overall Enteropathogen Prevalence

One hundred seven of 224 (47.8%) infants had at least 1 detectable enteropathogen and 39 (17.4%) had >1 pathogen detected (excluding Sabin viruses and rotavirus). Enteroaggregative *Escherichia coli* (EAEC) was the most prevalent pathogen (23.7% stools), followed by NPEV (13.0%), atypical enteropathogenic *E. coli* (6.7%) and Campylobacter (5.4%) (see Fig., Supplemental Digital Content 6; http://links.lww.com/INF/D658). At least 1 Sabin virus was detected in 30.0% of stools and rotavirus in 15.2%. Seventeen pathogens were present in <1% of samples and not included in individual pathogen analyses (see Table, Supplemental Digital Content 7, http://links.lww.com/INF/D659). Bacterial pathogens [mean: 0.42 (SD: 0.68) detected per sample] were more common than viral pathogens [0.26 (0.51) per sample]; few children had parasites [0.04 (0.20) per sample].

### Associations Between Enteropathogens and RVV Immunogenicity

**Figure 1.**
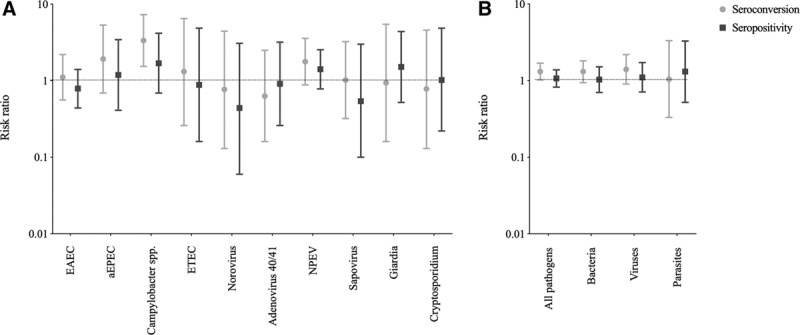
Associations between (A) individual pathogens and (B) grouped pathogens (grouped pathogen exposures do not include Sabin viruses and rotavirus) and oral rotavirus vaccine immunogenicity. Effect sizes and 95% confidence intervals shown are for the adjusted analysis. aEPEC indicates atypical enteropathogenic *Escherichia coli*; EAEC, enteroaggregative E. coli; NPEV, non-polio enterovirus.

Overall, there were few associations between enteropathogens and RVV immunogenicity. The prevalence of individual enteropathogens was generally low, and estimates were imprecise. Among enteropathogens with overall prevalence ≥1%, there were no significant associations between individual pathogens and rotavirus seroconversion or seropositivity in unadjusted analyses (see Table, Supplemental Digital Content 8, http://links.lww.com/INF/D660). After adjusting for prespecified variables, *Campylobacter* spp. was positively associated with rotavirus seroconversion [RR: 3.35; 95% confidence interval (CI): 1.54–7.29; Benjamini–Hochberg *P* = 0.008], but not with seropositivity (RR: 1.69; 95% CI: 0.69–4.17) or GMT (see Fig. 1 and Table, Supplemental Digital Content 8, http://links.lww.com/INF/D660).

There were no significant associations between pathogen burden (bacteria, viruses, parasites or all pathogens) and any measure of RVV immunogenicity (Fig. 1). Similarly, there was no effect of mixed infection (≥2 pathogen classes detected) on immunogenicity.

To further explore the relative contributions of NPEV versus polio, we undertook an analysis of pan-EV and Sabin quantity, by including both together in a regression model (see Fig., Supplemental Digital Content 9, http://links.lww.com/INF/D661). In the unadjusted and adjusted models, pan-EV quantity was positively associated with RVV seroconversion [adjusted RR 1.61 per 10-fold increase in pan-EV (95% CI: 1.35–1.91)]; in the same model, there was also a negative association between Sabin quantity and RVV seroconversion (adjusted RR: 0.76; 95% CI: 0.60–0.96) (Table 2). The direction and magnitude of these associations were similar for rotavirus seropositivity and GMT (Table 2).

**TABLE 2.**
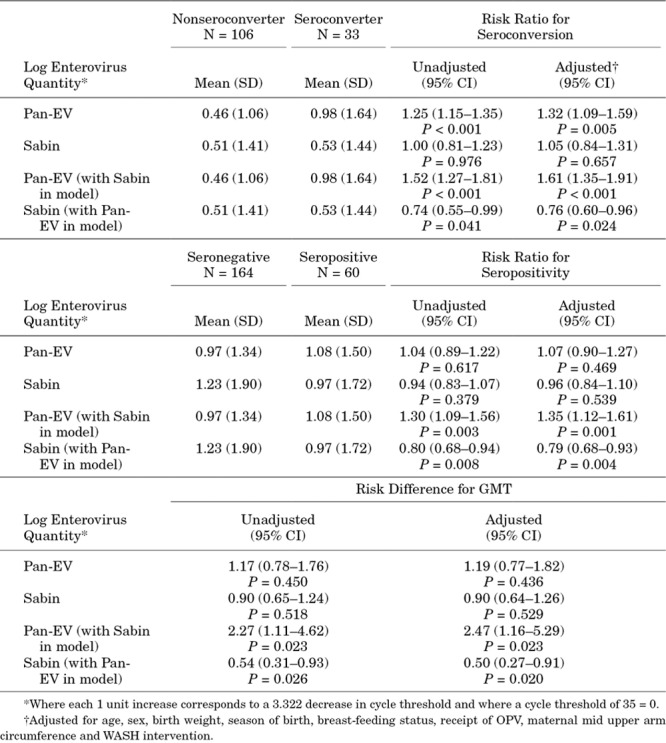
Associations Between Enterovirus Quantity (Pan-enterovirus and Sabin Viruses) and Oral Rotavirus Vaccine Immunogenicity

In a sensitivity analysis, restricting the timing of stool collection to 14 days prevaccination, there remained weak evidence of a positive association between NPEV and RVV immunogenicity (see Table, Supplemental Digital Content 10, http://links.lww.com/INF/D662). However, there were no other significant associations between stool enteropathogen detection and RVV immunogenicity. Findings were similar in further sensitivity analyses, increasing the sample size to include all infants with available stool from the first 6 months of life (see Table, Supplemental Digital Content 11, http://links.lww.com/INF/D663) or excluding EAEC from pathogen burden analyses (see Table, Supplemental Digital Content 12, http://links.lww.com/INF/D664), as in previous studies.^12^

### WASH Effects on Enteropathogen Prevalence

There were no meaningful differences between WASH and non-WASH groups in detection of any individual enteropathogen, or the overall number of pathogens (expressed either as total bacteria, total viruses, total parasites or all pathogens) (Table 3). Due to this low prevalence of detection, we did not go on to compare differences in pathogen quantity between WASH and non-WASH groups.

**TABLE 3.**
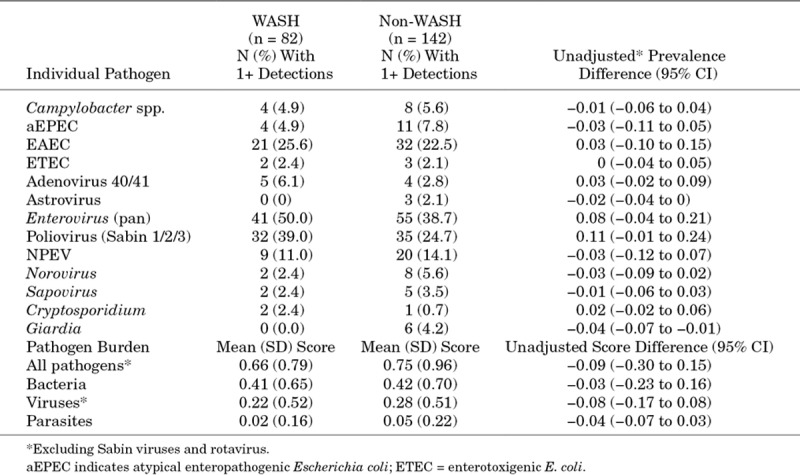
Differences in Individual Pathogen Prevalence and Pathogen Burden Associated With the WASH Intervention Among 224 Infants in the Substudy

## DISCUSSION

Using molecular diagnostic testing, enteropathogens were commonly detected around the time of oral rotavirus vaccination in rural Zimbabwean infants. However, we found no consistent associations between enteropathogens and RVV immunogenicity. Furthermore, improvements in household WASH did not reduce enteropathogen prevalence, suggesting that our previous finding of enhanced RVV immunogenicity among infants randomized to improved WASH is unlikely due to a reduction in enteropathogens around the time of vaccination.

Our data are consistent with recent studies^8,29^ reporting early and diverse enteropathogen exposure in low-income settings. For example, among nondiarrheal stool samples collected from Bangladeshi infants at 1 month of age, coinfections were highly prevalent (mean: 2.5 enteropathogens per stool).^30^ In our study, almost half of infants had at least 1 detectable pathogen and 17% had 2 or more pathogens in the first 3 months after birth, despite high rates of exclusive breast-feeding.^31^ By contrast, multiple enteric infections are rare in high-income settings.^30^

Some enteropathogens, particularly NPEVs, have previously been implicated in oral vaccine underperformance. In a meta-analysis of 25 trials, concurrent NPEV infection was associated with reduced OPV1 responses.^32^ A subsequent Indian study, using molecular diagnostic testing, showed significantly higher detection of NPEVs in nonresponders to OPV compared with responders.^10^ Associations between NPEV and rotavirus vaccination are more heterogeneous. Among Bangladeshi infants, pan-EV quantity was negatively associated with rotavirus seroconversion and breakthrough rotavirus diarrhea.^13^ Conversely, among Indian infants, EV prevalence did not differ significantly by rotavirus seroconversion status, while quantity was in fact greater among responders.^12^ In our study, we similarly found that the quantity of pan-EV was positively associated with RVV immunogenicity, after adjusting for Sabin quantity, across all 3 outcome measures (seroconversion, seropositivity and GMT). In the same model, Sabin quantity was negatively associated with RVV immunogenicity, which is in keeping with evidence that concurrent administration of OPV and RVV leads to lower seroconversion to RVV.^11,33^ We also found a positive association between NPEV detection and RVV immunogenicity (measured as seropositivity, but not seroconversion) when the analysis was restricted to children whose stool samples were collected 14 days before the first dose of Rotarix (see Table, Supplemental Digital Content 10, http://links.lww.com/INF/D662). Interpretation here is hindered by the small sample size and the imprecise subtractive logic, which uses pan-EV and Sabin qPCR assays to estimate NPEV and fails to account for coinfections. The latter may be particularly relevant since over 70% of our pan-EV positive samples contained at least 1 Sabin serotype, similar to the Indian study.^12^ Nevertheless, these observations provide an intriguing, albeit inconsistent, signal that EV detection close to the first dose of rotavirus vaccination is associated with immune responses to RVV. The inconsistency may be attributable to different EV strains having distinct interactions with RVV, resulting in geographic variation. Next-generation sequencing would help discriminate between strains to better understand the precise relationship between EVs and RVV performance.

The relationship between other enteric pathogens and RVV immunogenicity also remains uncertain. In Bangladesh, there was no association between nonviral enteric pathogens and RVV seroconversion.^13^ In India, RVV responses were increased among infants with detection of at least 1 bacterial pathogen,^12^ although this effect was contingent on the omission of EAEC (detectable in 47.9% of 6-week-old infants) from their analyses. Overall, we found no correlation between bacterial burden and RVV immunogenicity, irrespective of EAEC inclusion (Table S8). In adjusted analyses, *Campylobacter* spp. were associated with increased vaccine seroconversion. However, we did not observe a positive association between *Campylobacter* and rotavirus seropositivity or GMT, and these inconsistent findings suggest that the association is unlikely to be meaningful.

We recently reported that household improvements in WASH led to increased rotavirus immunogenicity.^14^ However, we found no evidence that WASH reduced enteropathogen prevalence around the time of vaccination, so this is unlikely to explain our results. It is possible that WASH had an impact on other intestinal factors, such as environmental enteric dysfunction^34^ or microbiota dysbiosis,^35^ which have both been implicated in oral vaccine failure. We previously showed that non-WASH infants in SHINE had higher baseline rotavirus seropositivity, suggesting more wild-type rotavirus infection before vaccination at 6 weeks of age. The current study used molecular methods to detect rotavirus in stool (rather than immune responses to the virus). Among infants with specimens available in the 2 weeks prevaccination, detection of rotavirus was too seldom to make meaningful comparisons (4.2% non-WASH vs. 0% WASH infants). It remains plausible that the WASH intervention (which began antenatally) reduced neonatal rotavirus infection and thereby improved immune responses to the vaccine, although current dogma is that WASH does not substantially reduce rotavirus transmission. Further studies, using molecular assays to distinguish wild-type rotavirus from Rotarix-specific nonstructural protein 2, are needed to explore this in more detail.^36^

Our study has several strengths. This was a well-characterized cohort of infants from a large, community-based, cluster randomized trial. The alarmingly low level of RVV seroconversion (23.7% overall) suggests that the study population is suitably representative of children in whom oral vaccines are poorly efficacious. To our knowledge, this is the first study in Africa to evaluate associations between enteropathogens and RVV immunogenicity, and we used sensitive molecular detection methods. However, the study also has several limitations. First, it remains unclear how rotavirus-specific IgA titers relate to protection in low-income settings, although it remains the best available measure of immunogenicity.^37^ Second, our ability to detect associations between enteropathogens and vaccine immunogenicity was reduced by low rates of seroconversion and a relatively small sample size because we restricted the inclusion of stool samples to a narrow window around the time of rotavirus vaccination. However, our inferences remained unchanged in sensitivity analyses which liberalized the window for stool collection. Finally, the low prevalence of some pathogens means that the magnitude of exposure was difficult to compare between infants. However, it is unlikely that pathogens detected in such small quantity contribute to the substantial oral vaccine efficacy gap.

Despite frequent pathogen detection around the time of vaccination, we found few consistent associations between enteropathogens and RVV immunogenicity. There was no evidence that a household-level WASH intervention reduced enteropathogens, meaning our previous finding of improved RVV immunogenicity with WASH is unlikely due to reduced pathogen exposure. More mechanistic studies are needed to better understand the interplay between the intestinal environment and oral vaccine responses.

## ACKNOWLEDGMENTS

The authors thank all the mothers, babies and their families who participated in Sanitation Hygiene Infant Nutrition Efficacy. The authors gratefully acknowledge the leadership and staff of the Ministry of Health and Child Care in Chirumanzu and Shurugwi districts and Midlands Province (especially environmental health, nursing and nutrition) for their roles in operationalization of the study procedures. The authors acknowledge the Ministry of Local Government officials in each district who supported and facilitated field operations.
